# Abcès hépatiques à pyogènes secondaires à une perforation gastrique par un corps étranger compliquée de péritonite aiguë : à propos d'un cas à l'Hôpital Principal de Dakar, Sénégal

**DOI:** 10.48327/mtsi.v4i1.2024.390

**Published:** 2024-02-12

**Authors:** Patrick AYONGA NDEBA, Yvette AKONKWA, Fatimata WONE, Sihem GOURARI

**Affiliations:** 1 Université Cheikh Anta Diop (UCAD), Faculté de médecine, pharmacie et odontologie, Dakar, Sénégal; 2 Service des maladies infectieuses et tropicales, Centre hospitalier universitaire national Fann (CHUNF), Dakar, Sénégal; 3 Service d'hépato-gastro-entérologie et chirurgie digestive, Hôpital Principal de Dakar (HPD), Dakar, Sénégal; 4 Département de médecine interne et maladies infectieuses, Pavillon Boufflers, Hôpital Principal de Dakar (HPD), Dakar, Sénégal; 5 Service de médecine interne et maladies infectieuses, Centre hospitalier de Périgueux, Périgueux, France

**Keywords:** Abcès hépatique, Corps étranger, Arête de poisson, Perforation gastrique, Drainage, Hôpital, Dakar, Sénégal, Afrique subsaharienne, Hepatic abscess, Foreign body, Fishbone, Gastric perforation, Drainage, Hospital, Dakar, Senegal, Sub-Saharan Africa

## Abstract

L'ingestion accidentelle d'un corps étranger dans le tractus gastro-intestinal n'est pas exceptionnelle, cependant le développement d'un abcès hépatique secondaire à une perforation digestive par un corps étranger est rare. Nous rapportons le cas d'abcès hépatiques à pyogènes secondaires à une perforation gastrique par une arête de poisson compliquée d'une péritonite aiguë. Un patient de 53 ans a été admis dans notre hôpital avec comme principales plaintes : douleurs abdominales diffuses avec vomissements dans un contexte de fièvre et d'asthénie physique. Une hépatomégalie fébrile douloureuse avec ictère a été objectivée, ainsi qu'un syndrome inflammatoire biologique non spécifique. Une tomodensitométrie initiale abdomino-pelvienne a révélé des abcès hépatiques multifocaux. Devant l’échec thérapeutique initial associant une antibiothérapie parentérale et drainage des abcès, une seconde tomodensitométrie abdominale a permis d'identifier un corps étranger à cheval entre la paroi antropylorique et le segment I du foie. Une laparotomie médiane xypho-pelvienne a été réalisée avec issue du liquide péritonéal de près de 200 cc louche. Une arête de poisson d'environ 5 cm de long a été extraite par laparotomie, suivie d'une suture gastrique avec épiplooplastie, une toilette péritonéale et un drainage. Un traitement adjuvant symptomatique a été initié, dont un inhibiteur de la pompe à protons (pantoprazole). Le patient a également bénéficié d'un support transfusionnel devant l'anémie. L'antibiothérapie a été poursuivie pendant une durée totale de 2 semaines après la chirurgie. L’évolution a été favorable avec une imagerie de contrôle à 3 mois, montrant une résorption complète des abcès hépatiques.

## Abstract (en)

Accidental ingestion of a foreign body into the gastrointestinal tract is not uncommon, however the development of hepatic abscesses secondary to digestive perforation by a foreign body is rare. We report the case of pyogenic hepatic abscesses secondary to gastric perforation by a fishbone complicated by acute peritonitis. A 53-year-old patient was admitted to our hospital with the main complaints: diffuse abdominal pain with vomiting in a context of fever and physical asthenia. A painful febrile hepatomegaly with jaundice was objectified, as well as a non-specific biological inflammatory syndrome. An initial abdominopelvic CT scan revealed multifocal liver abscesses. Faced with the initial therapeutic failure associating parenteral antibiotic therapy and abscess drainage, a second abdominal CT scan identified a foreign body straddling the antropyloric wall and segment I of the liver.

A xypho-pelvic midline laparotomy was performed with nearly 200 cc of peritoneal fluid coming out. A fishbone approximately 5 cm long was extracted by laparotomy, followed by gastric closure with omentum, peritoneal cleansing and drainage. Symptomatic adjuvant treatment was initiated, including a proton pump inhibitor (Pantoprazole). He also benefited from transfusion support in the face of anemia. Antibiotic therapy was continued for a total of 2 weeks after surgery. The evolution was favorable with follow-up imaging at 3 months, showing complete resorption of the hepatic abscesses.

## Introduction

Les abcès hépatiques sont des affections rares et graves, dont l'incidence augmente ces dernières années [11]. La morbidité associée à cette affection est importante, avec un risque de mortalité compris entre 6 et 10 % [16]. L'agent pathogène est le plus souvent bactérien responsable d'abcès à pyogènes. Il peut être exceptionnellement parasitaire ou fongique [9]. L'incidence annuelle des abcès à pyogènes diffère d'une région à une autre, néanmoins elle est estimée entre 1,1 et 2,3 cas pour 100 000 personnes [5, 10, 15, 16]. L'origine est principalement biliaire ou portale [9]. Il est cependant rare de rencontrer le développement d'un abcès du foie secondaire à une pénétration d'un corps étranger au niveau du tube digestif. Nous rapportons la prise en charge d'un cas d'abcès hépatiques à pyogènes secondaires à une péritonite à la suite d'une perforation gastrique par un corps étranger.

## Observation

**Figure 1:**
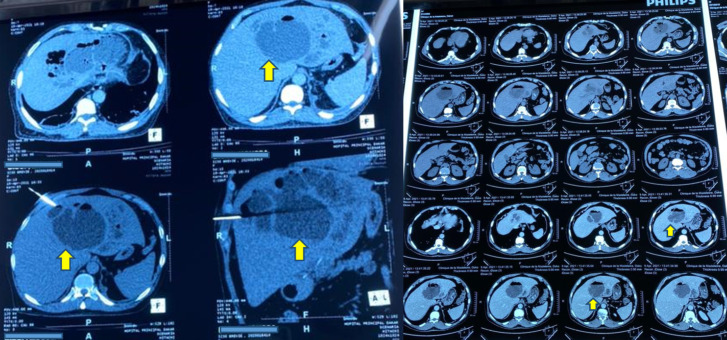
Clichés de tomodensitométrie abdomino-pelvienne d'un patient âgé de 53 ans qui présente des douleurs abdominales aiguës avec hépatomégalie et sepsis, montrant des hypodensités au niveau du lobe gauche du foie à contenu hydro-aérique (flèches jaunes) Abdominopelvic computed tomography images of a 53-year-old patient presenting with acute abdominal pain with hepatomegaly and sepsis, showing hypodensities in the left lobe of the liver with water-aeric content (yellow arrows)

Il s'agissait d'un patient âgé de 53 ans, sans antécédent pathologique particulier ni notion de phytothérapie, suivi depuis 3 ans pour une maladie de Biermer sous Vitamine B12 qui a été reçu en urgence pour douleurs abdominales diffuses avec vomissements postprandiaux, fièvre vespérale et asthénie physique intense, d’évolution aiguë. Pas de notion de diarrhée ni de retard des selles rapportée. L'examen physique retrouvait un abdomen légèrement ballonné, avec une hépatomégalie douloureuse à la palpation et une sensibilité épigastrique avec défense, un ictère et un indice de performance de l'Organisation mondiale de la santé de grade 3, le tout dans un contexte de fièvre chiffrée à 38,6 - 39 °C avec un bon état hémodynamique. Aucun autre point d'appel clinique évident n'a été identifié. Une tomodensitométrie abdomino-pelvienne (TDM AP) réalisée en urgence a révélé des abcès hépatiques multifocaux (88 × 76 × 72 mm pour les segments II et IVa; 22 à 27 mm pour les segments I et III), avec une lésion dominante à cheval sur les segments II et IVa (Fig. 1).

Les résultats des examens de laboratoire sont résumés dans le Tableau I.

**Tableau I:** Résultats des examens de laboratoire d'un patient avec abcès hépatiques Laboratory test results of a patient with hepatic abscesses

Examen (référence normale)	Résultat
Hémoglobine (12,5 – 18 g/dl)	8,6
Leucocytes (4,00 – 10,00 109/1)	20,480
Thrombocytes (150 – 450 109/1)	630,000
Bilirubine totale (1,0 – 14,0 mg/1)	79,2
Albumine sérique (37,0 – 53,0 g/1)	11,0
Transaminase ALAT (SGPT) (10 – 40 Ul/l)	157
Transaminase ASAT (SGOT) (10 – 31 Ul/l)	80
Phosphatases alcalines (50 – 136 Ul/l)	291
Gamma-glutamyltransférase (GGT) (15 – 85 Ul/l)	768
Urée (19 – 44 mg/dl)	21
Créatininémie (0,73 – 1,18 mg/dl)	0,98
Kaliémie (3,5 – 4,5 mmol/l)	3,6
Natrémie (135 – 145 mmol/l)	143
PSA total (0,00 – 5,00 ng/ml)	2,67
Protéine C réactive (CRP), (inférieur à 5,00 mg/l)	227,50
Alpha-fœtoprotéine (inférieur à 7 ng/l)	3
Antigène carcino-embryonnaire (ACE) (inférieur à 5 pg/l)	0,94
Glycémie à jeun (0,74 – 1,09 g/l)	0,97
Taux de prothrombine (TP) (70 – 100 %)	50
Sérologie rétrovirale (VIH)	Négative
Antigène HBs	Négatif
Anticorps anti-VHC	Négatif
Goutte épaisse	Négative
Sérologie amibienne	Négative (à 2 reprises)

Le traitement initial a consisté en un drainage scanno-guidé des abcès et une antibiothérapie empirique intraveineuse faite de ceftriaxone 2 g par jour, de gentamicine 3 mg/kg (soit 180 mg pour 60 kg de poids) une fois par jour pendant 3 jours et de métronidazole 500 mg toutes les 8 heures, associée à des mesures adjuvantes.

L'analyse microbiologique du pus prélevé lors du drainage, a montré de nombreux polynucléaires neutrophiles et la présence des rares bacilles Gram négatif avec quelques cocci à Gram positif et l'absence d’éléments fongiques à la coloration Gram. Une première culture réalisée étant non contributive, une seconde analyse effectuée 72 heures après était stérile. Deux hémocultures réalisées étaient négatives. L’évolution a été marquée par la persistance du tableau clinique avec aggravation des douleurs abdominales diffuses, plus marquées à l’épigastre et à l'hypochondre droit.

**Figure 2:**
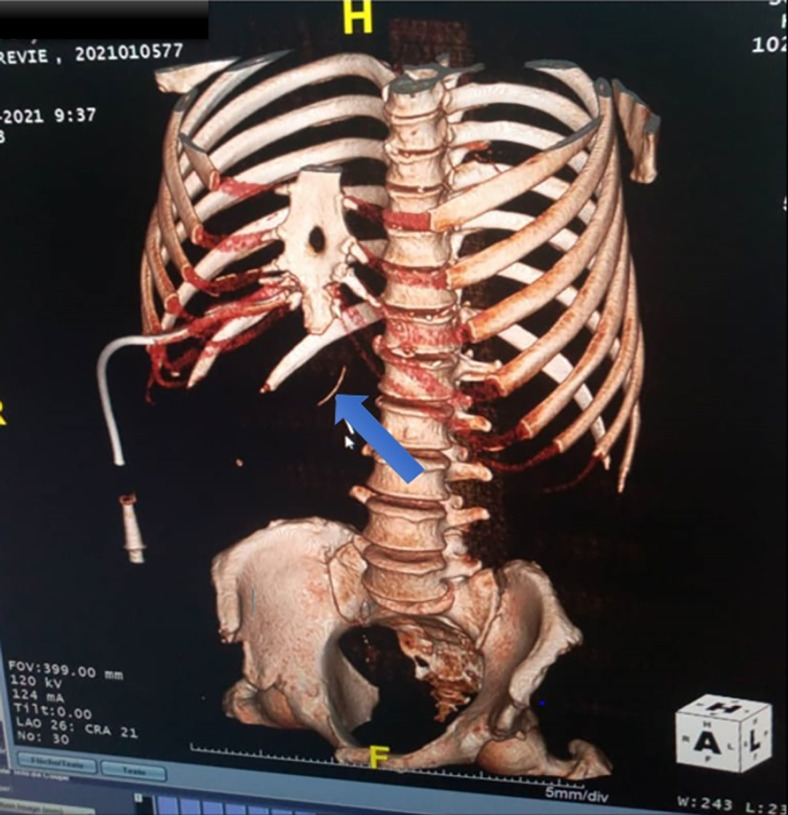
Tomodensitométrie abdominale en reconstruction 3D montrant un corps étranger (flèche bleue) Abdominal computed tomography in 3D reconstruction showing the foreign body (blue arrow)

Ceci a motivé la réalisation d'un second scanner thoraco-abdomino-pelvien. Celuici a montré la persistance d'une collection hépatique de 64,8 × 53 mm, multi-cloisonnée au lobe hépatique gauche et une collection résiduelle au lobe droit de 43 mm. À la relecture des images scannographiques, un corps étranger a été identifié à cheval entre la paroi antro-pylorique et le segment I du foie. De plus, un épanchement péri-hépatique de la fosse iliaque droite et du Douglas avec infiltration de la graisse péritonéale (avec intégrité des autres viscères pleins) a été également objectivé (Fig. 2 et 3). Il n'y avait pas de lésion vasculaire apparente.

**Figure 3:**
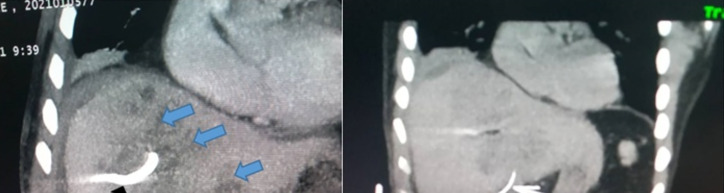
Tomodensitométrie abdominale montrant des abcès hépatiques multifocaux, hypodensité mal limitée (flèches bleues), avec drain transcutané en place (flèche noire) et le corps étranger (flèche jaune) Abdominal computed tomography showing multifocal hepatic abscesses, poorly limited hypodensity (blue arrows), with transcutaneous drain in place (black arrow) and the foreign body (yellow arrow)

Une laparotomie médiane xypho-pelvienne a été réalisée avec évacuation d'environ 200 cm^3^ de liquide pérotonéal louche. L'exploration per-opératoire a permis d'objectiver une perforation gastrique en pré-pylorique d'environ 0,5 cm de diamètre avec présence d'un corps étranger, type « arête de poisson », de près de 5 cm de long. S'en est suivie une extraction de l'arête de poisson, une suture gastrique avec épiplooplastie, une toilette péritonéale et un drainage (Fig. 4 et 5).

**Figure 4:**
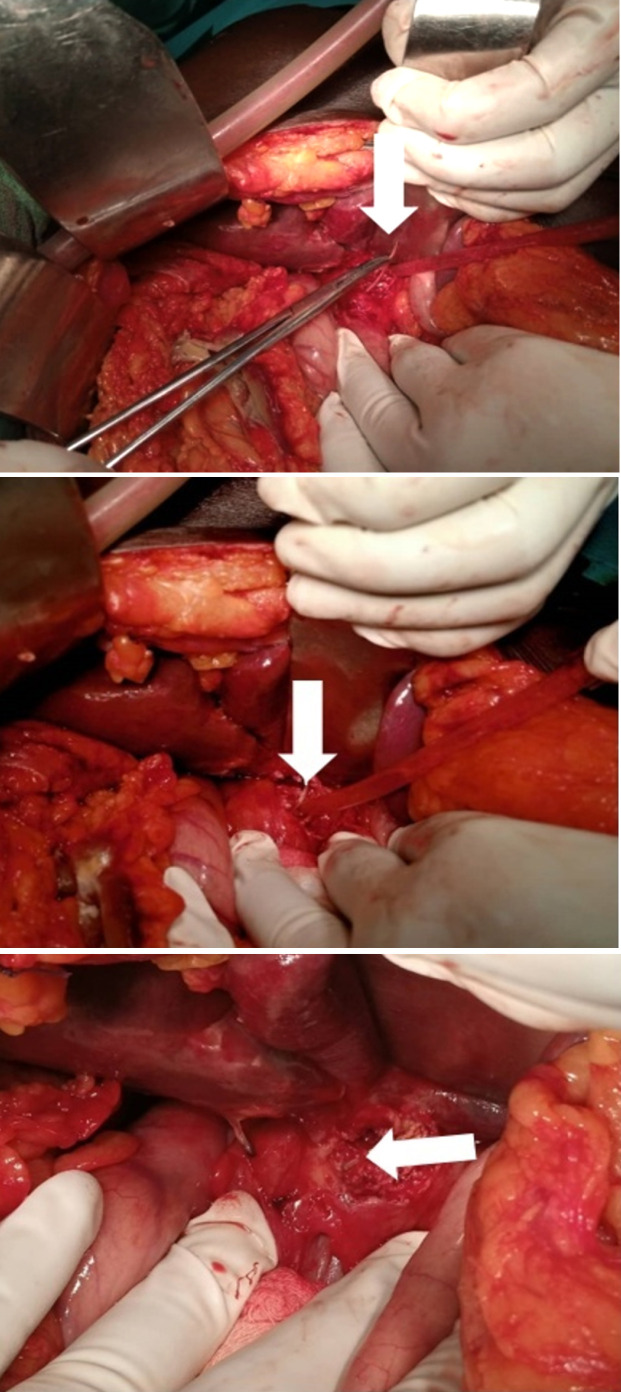
Vue per-opératoire de l'extraction du corps étranger avec aspect macroscopique (flèches blanches) Intraoperative view of foreign body extraction with macroscopic aspect (white arrows)

**Figure 4:**
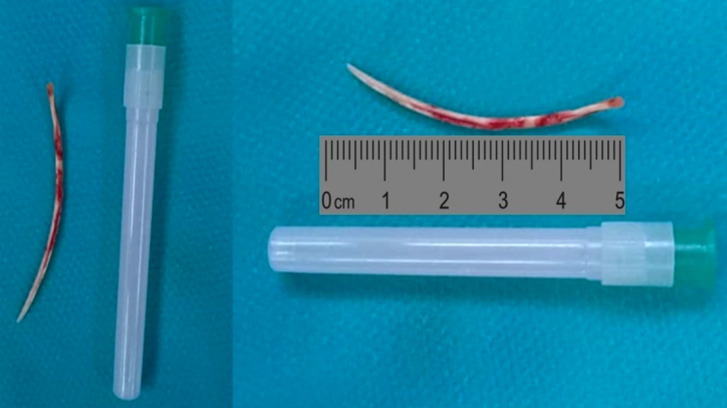
Vue macroscopique du corps étranger extrait : une « arête de poisson » d'environ 5 cm de long Macroscopic view of the extracted foreign body: a “fishbone” about 5 cm long

L’évolution au 7^e^ jour post-opératoire était marquée par une suite opératoire simple et une nette amélioration clinico-biologique. L'antibiothérapie a été poursuivie pendant une durée totale de 2 semaines après la chirurgie sans récidive des abcès. Le patient a été revu en consultation externe 15 jours après son retour à domicile sans aucune particularité clinico-biologique. L'imagerie de contrôle à 3 mois a montré une résorption complète des abcès hépatiques.

## Discussion

Il existe très peu des données décrivant l'incidence des cas d'ingestion de corps étrangers parmi la population adulte. Les corps étrangers ingérés (arête de poisson, curedents, os de poulet, objets métalliques, etc.) ne sont cependant pas exceptionnels, mais des abcès hépatiques secondaires à la migration d'un corps étranger sont rares [18]. Ils doivent être pris en compte ou être recherchés systématiquement en cas d'antécédent d'ingestion de corps étranger, ou lorsque l'imagerie est évocatrice ou encore en cas d’échec thérapeutique malgré un drainage et une antibiothérapie appropriée. Par contre, seulement 5 % des patients présentant un abcès du foie secondaire à l'ingestion d'un corps étranger se souviennent de l'ingestion [14, 18]. Le patient avait mentionné plus tard, à la suite de la découverte du corps étranger au scanner abdominal et en post-opératoire, qu'il consommait régulièrement du poisson, mais ne se souvenait pas avoir avalé une arête. Devant un abcès hépatique à pyogènes, les principales voies de contamination à évoquer sont entre autres (par ordre de fréquence) : biliaire, portale, artérielle, par contiguïté et/ ou traumatique, et cryptogénétique (si aucun foyer d'origine n'est retrouvé) [5, 9, 10, 15]. Les traumatismes pénétrants du foie, à l'origine d'un abcès hépatique, peuvent parfois être discrets, causés par l'ingestion d'un corps étranger comme une arête de poisson [18], comme dans le cas de notre observation.

Du point de vue physiopathologique, dans près de 80 à 90 % des cas, les corps étrangers ingérés traversent le tractus gastro-intestinal sans incident en 1 semaine. Dans le cas contraire, ils sont généralement secondaires à une obstruction ou à une perforation, qui surviennent chez moins de 1 % des patients [14, 18]. Ces perforations peuvent survenir à n'importe quel endroit du tractus gastro-intestinal, mais les sites les plus courants sont localisés au niveau de l’œsophage, de l'appendice iléo-cæcal et du recto-sigmoïde [14, 18]. La survenue d'une péritonite généralisée secondaire à la rupture d'un abcès hépatique due à la pénétration d'une arête de poisson n'a été que très peu documentée. Le développement d'un abcès hépatique à pyogènes comme complication de l'ingestion d'un corps étranger a rarement été rapporté. Un premier cas avait été mentionné par Lambert en 1898 [12]. Dans une revue de la littérature anglaise, seuls 9 cas d'abcès hépatiques secondaires à la pénétration d'arêtes de poisson ont été rapportés [14]. Et depuis lors, plusieurs autres rares cas ont été documentés [1, 3, 4, 6, 13]. En effet, les abcès hépatiques peuvent résulter de la dissémination hématogène d'agents pathogènes ***via*** le système veineux porte à partir du tractus gastro-intestinal ou de l'artère hépatique du fait d'une septicémie, d'une cholangite ascendante ou d'une propagation locale de l'infection [9]. Le développement de l'abcès hépatique dans ce cas a probablement été causé par la persistance et la propagation de l'infiltration péri-hépatique.

Les manifestations cliniques de l'abcès hépatique secondaire à la perforation par un corps étranger sont variables et généralement discrètes. Les principaux symptômes sont peu spécifiques : fièvre isolée, altération de l’état général, douleur abdominale diffuse, nausées ou vomissements, diarrhée, douleur thoracique, toux sèche, sueurs nocturnes. Seulement 10 % des patients se présentent avec la triade classique dite de Fontan, associant fièvre, hépatomégalie et douleur de l'hypocondre droit [18]. Le patient a présenté également un ictère avec un taux de bilirubine totale supérieure à 5 fois la normale. En général, l'ictère est rare, mais sa présence témoigne habituellement d'une angiocholite associée ou d'une atteinte hépatique diffuse (dont syndrome de cytolyse et cholestase) en cas d'abcès multiples [14, 18].

L’échographie et la tomodensitométrie se sont révélées très utiles dans le diagnostic [2, 11, 14]. L’évolution clinique du patient était aspécifique et ses antécédents médicaux ne fournissaient aucune information sur l'ingestion d'un corps étranger. Nous avons pu établir un diagnostic précis en préopératoire sur la base des résultats du scanner.

La plupart des abcès du foie sont polymicrobiens. L'examen bactériologique du pus est généralement positif dans 80 % des cas, mettant souvent en évidence entre 2 et 4 germes (bacilles Gram négatif, entérocoques, anaérobies). Les abcès multiples sont plus fréquemment polymicrobiens que les abcès uniques. Les hémocultures sont positives dans 25 à 50 % des cas, à condition de réaliser des prélèvements multiples (aérobies et anaérobies) avant toute administration d'antibiotiques [5, 10, 11, 15]. Dans notre observation, toutes les cultures sont restées stériles, probablement décapitées par l'antibiothérapie précoce. Par contre, en cas d'abcès cryptogénétique, les hémocultures et les prélèvements de pus sont le plus souvent négatifs [11].

Le traitement consiste en un drainage de l'abcès, l’élimination du corps étranger et une antibiothérapie adaptée [7, 19]. Les taux de guérison sans extraction, en cas d'abcès hépatique secondaire à un corps étranger, sont extrêmement faibles. D'où l'intérêt, en plus de l'antibiothérapie, de réaliser l'extraction du corps étranger soit par une laparotomie ou une laparoscopie, soit une endoscopie du tractus gastro-intestinal supérieur ou inférieur [7, 8, 17, 19]. Le patient a bénéficié d'une laparotomie en urgence avec extraction de l'arête de poisson, d'une toilette péritonéale et d'un drainage, associés à une antibiothérapie probabiliste à large spectre visant les bactéries Gram positif et Gram négatif (en particulier les anaérobies).

L’évolution sous traitement est le plus souvent favorable [17]. Le taux de létalité dans les différentes séries varie entre 10 et 20 %. Il est le plus élevé en cas de traitement antibiotique seul et le plus bas en cas de traitement percutané (drainage, ponction) associé à l'antibiothérapie. Dans le cas le plus fréquent d'une évolution favorable sous traitement, un scanner de contrôle doit être réalisé 1 à 2 mois après l'arrêt de l'antibiothérapie. Le taux de récurrence est relativement faible : 7 % en cas de traitement percutané, 9 % en cas de drainage chirurgical, et 10 % en cas d'antibiothérapie seule [7, 8, 17, 19].

## Conclusion

Le développement d'un abcès hépatique secondaire à une pénétration d'un corps étranger au niveau du tube digestif est rare. L’établissement d'un diagnostic préopératoire est difficile, car la plupart des patients ne se souviennent pas d'avoir avalé le corps étranger. Ainsi, la présence d'un corps étranger doit être évoquée après l’élimination des autres causes fréquentes et devant la persistance du tableau clinique ou la récidive des abcès. Une lecture attentive de l'imagerie disponible recherchant systématiquement un corps étranger est nécessaire.

## Contributions des auteurs

Patrick AYONGA NDEBA : conception et rédaction du manuscrit.

Yvette AKONKWA, Fatima WONE, Sihem GOURARI : discussion et relecture du manuscrit.

## Liens d'intérêts

Les auteurs ne déclarent aucun conflit d'intérêts.
